# A new integrated approach for adolescent health and well-being: the AVATAR project

**DOI:** 10.1186/s12955-020-01291-6

**Published:** 2020-03-18

**Authors:** Francesca Mastorci, Luca Bastiani, Gabriele Trivellini, Cristina Doveri, Cristina Vassalle, Alessandro Pingitore

**Affiliations:** 1 Clinical Physiology Institute, CNR, Via Moruzzi, 1, 56124 Pisa, Italy; 2 Fondazione G. Monasterio - Regione Toscana, Via Moruzzi, 1, 56124 Pisa, Italy

**Keywords:** Adolescent, Health, Well-being, Lifestyle, Social context, Emotions, Cognitive abilities

## Abstract

**Background:**

Limited number of studies examined the relationship between factors (lifestyle, social, emotional, cognitive) affecting adolescents’ health and well-being. The aims of this study were to identify the more important variables of the different components affecting adolescents’ health [lifestyle habits (LH); emotional status (ES); social context (SC); and cognitive abilities (CA)], and explore the relationship between the aforementioned components.

**Methods:**

Data were collected between 2017 and 2018 from 756 eligible students, recruited from 5 Italian junior high school**,** by using KIDSCREEN-52 and cognitive processing using the Stroop Test. School engagement was estimated through questions concerning the scholastic achievement.

**Results:**

Of 756 adolescents, 395 were boys with a mean (SD) age of 12.19 (0.81) years. Compared to International T-value of reference group for KIDSCREEN-52, autonomy, bullying, psychological well-being and mood were lower than the reference groups, while self-perception score was higher. For LH, the most important predictor was autonomy (***p*** < .0001). The most important aspects in the SC were the relationship with the parents (***p*** < .0001), and the adolescent’s relationships with peers (***p*** < .0001). For ES, mood variables had the greatest contribution (***p*** < .0001). The School performance related to Language & Literature (p < .0001) was the most important predictor in the CA latent variable. LH was positively associated with SC (*p* < .0001), ES (p < .0001), and CA (*p* < .0001). SC was positively associated with ES (p < .0001) and with CA (p < .0001).

**Conclusions:**

**T**his study suggests the importance of an integrated approach to characterize adolescents’ health and well-being. The approach suggested here may highlight additive synergistic effects of the various components in health and well-being assessment that may not be considered with a late approach and focused only on single factors.

## Background

In the last decades, we have witnessed a substantial change in definition of health, from absence of disease to a state of well-being, intending adolescence as a period when potentially deleterious behaviours begin [1]. Adolescents are central to every major current challenge in global health compared to that of children and adults, due to their fast physical, intellectual, and emotional development [2]. Despite health services targeting adolescents are highly fragmented, poorly coordinated, and uneven in quality, adolescence is a heightened period of vulnerability, specifically because of gaps between emotion, cognition, and behaviour, as part of their natural biological and social transition [3]. Adolescence is considered a time of good health when disease burden is low [4], however, epidemiological data showed that, behavioural risk factors may have crucial effects later in life [1]. This highlights the importance to develop preventive approaches and strategies contributing to maintain or improve health status and reducing predisposition to chronic degenerative diseases in adulthood [5]. For this purpose, nowadays, different indicators for adolescent health, belonging to components of psychosocial, emotional, cognitive and lifestyle, have been identified [6]. However, they have been considered individually and not in an integrated network.

The approach suggested here may highlight additive synergistic effects of the various components in health and well-being that may not be considered when focused only on single factors.

Aim of the study was to identify the principal variables of the different components affecting adolescents’ health (i.e. lifestyle habits, social context, emotional status, cognitive abilities), and explore the relationship between the aforementioned components, assuming that these are not independent to each other, according to a multifactorial and multisectorial perspective.

## Methods

### Study participants

Between 2017 and 2018, 785 adolescent students were enrolled from five junior high schools across Italy, according to the following inclusion criteria: age 10–14 years, absence of neuropsychiatric or other diseases, informed consent signed, and filling of the entire questionnaires proposed. Among these, 5 were excluded for diagnosed neuropsychiatric or other diseases, 10 who did not sign informed consent, and 14 who did not fill all the questionnaires. Therefore, the final population consisted of 756 adolescents (mean age 12.19, male 393). In every school class, all the adolescents filled out the questionnaire, and those who were not eligible due to exclusion criteria reasons were excluded from the study retrospectively.

All the schools joined the AVATAR project, that is the acronym for “A new purpose for promotion and eVAluation of healTh and well-being Among healthy teenageRs”. AVATAR project aim is to develop a new tool to assess lifestyle habits, social context, emotional status, and mental skills in adolescents, and to define an integrated index of the best indicators of well-being [7].

All students belonged to the AVATAR school Italian network called Rete Ulisse - Schools joining scientific research and educational innovation, in order to guarantee the heterogeneity of social, cultural, socioeconomic, and geographical background.

Participants were previously instructed on how to fill out the questionnaires and how to conduct the tests (see Data collection section for more details). All tests were conducted during participants’ computer lesson in school time. No incentive was provided to adolescents or parents. A research assistant was available to provide information and technical support to complete questionnaires. All parents or legal guardians gave informed consent, and authorized researchers to use their data in accordance with Italian law. All procedures performed in the study were in accordance with the ethical standards of the institutional and/or national research committee and with the 1964 Helsinki declaration and its later amendments or comparable ethical standards.

### The AVATAR methodology

The proposed multifactorial approach is focused on the integration of four components of health-related well-being [lifestyle habits (LH); emotional status (ES); social context (SC); and cognitive abilities (CA)], as perceived by adolescent, and how they relate to each other.

Conceptually, the AVATAR methodology is based on a multidimensional construct, covering physical, emotional, mental, social, and cognitive components of well-being and functioning as perceived by adolescents, developed within the AVATAR project (see [7] for more details on the used construct).

Specific indicators were selected according to the analysis of the existing literature in adolescent’s health and well-being [5, 6, 8].

**Fig. 1:**
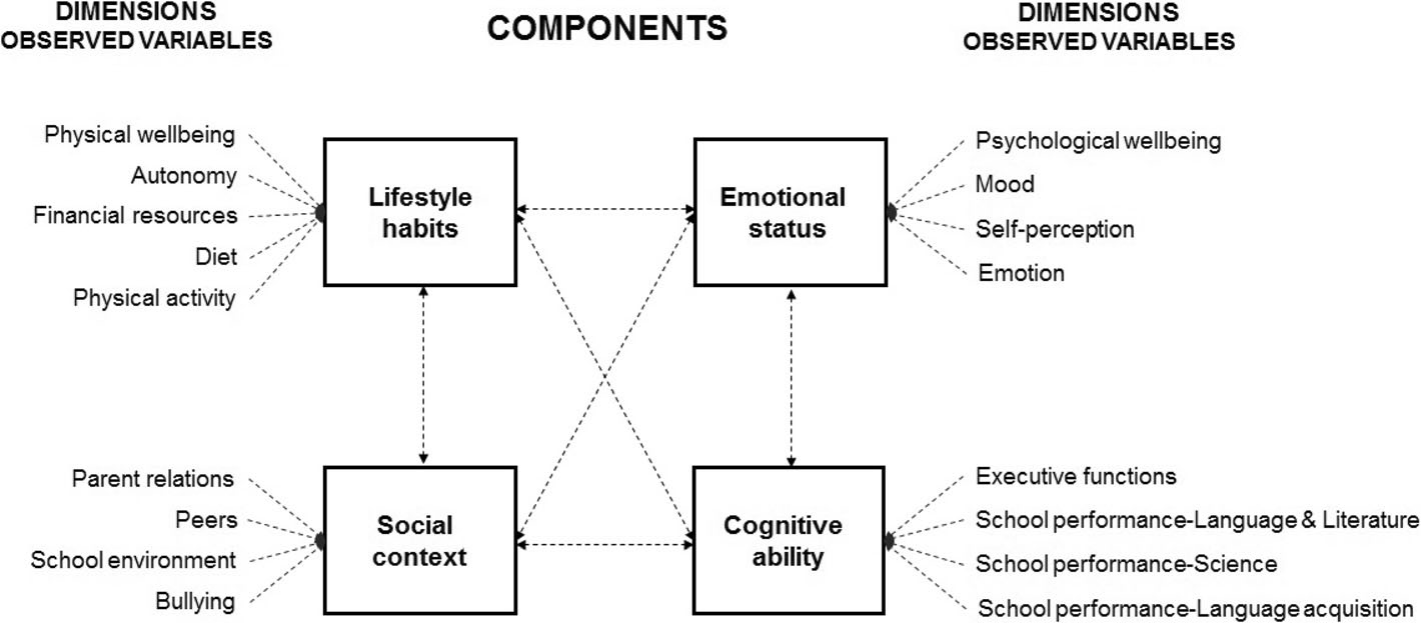
AVATAR methodology. Dotted lines are described in the Structural Model (Fig. 2)

Figure 1 depicts the single variables, namely observed variables, for each component.

### Data collection

Data were collected with AVATAR Web-tool [7]. A socio-demographic data record was used to collect information on gender, age, schooling, family structure, and body mass index, according to WHO age group [9]. The Italian version of KIDSCREEN-52 was used to assess health-related quality of life [10, 11]. The KIDSCREEN is a self-report questionnaire designed to address health-related quality of life, aimed to monitor and measure the personal experiences in children and adolescent about their perception of health status and well-being. The questionnaire, that describes physical, psychological, mental, social, and functional aspects of well-being, consists of 52 items grouped in 10 dimensions [10, 11].

KIDSCREEN questionnaires has been psychometrically tested using data obtained in a multicentre European study which included a sample of 22,827 children recruited in 13 countries [12].

In addition, physical activity and dietary intake food frequency per week (e.g. meat, vegetables, fish) were considered. The cognitive processing was evaluated with the Stroop Color and Word Test Children’s Version [13]. This test, standardized for the identification of executive function deficits in children and adolescent, evaluates the Stroop performance as measure of ability for planning, directing and maintaining attention, organization, abstract reasoning and problem-solving, self-regulation, and motor control.

The school engagement has been estimated through questions concerning the scholastic achievement in Language & Literature, Language acquisition, and Science.

### Statistical analysis

All statistical analyses were completed using Stata/SE 13.1 and SPSS Version 24. Categorical variables were expressed as percentages, and continuous variables were expressed as mean and standard deviation (SD) and mean and standard error for standardized regression coefficient for the Structural Model (Table 3). First, descriptive analyses were conducted to describe the sample. Pearson’s correlation coefficients were calculated to examine relationships between continuous variables. The level of significance was set at *p* < .05.

Structural equation modeling (SEM) using Stata/SE 13.1, used to test the proposed model (Fig. 1) [14, 15]. The path analysis technique used measures to the extent that the model fit a data set and allowed testing of interrelationships between several variables simultaneously.

The confirmatory factor analysis was used to test an overall measurement model that included five correlated latent variables. Overall model fit was assessed using different statistics. First, a chi-square analysis was used. The other indices were the Root Mean Square Error of Approximation (RMSEA) (values between 0.05 and 0.08 indicate acceptable fit, and values < 0.05 a good fit), Comparative Fit Index (CFI) (values > 0.90 indicate reasonable fit, > 0.95 good fit), and Standardized Root Mean Square Residual (SRMR) (values < 0.10 indicate good fit) [16]. The measurement model was first tested to ensure that each of the observed variables was a sufficient indicator of the hypothesized latent variables. Next, the model including the hypothesized pathways was evaluated.

## Results

### Demographic and health-related quality of life information

Participants consisted of 756 students with average age of 12.19 years (0.81). The number of males was 395 (52%), while females were 360 (48%). The whole sample had normal weight with BMI average of 19.2 Kg/m^2^ (4.41).

Health-related quality of life variables, expressed as mean T scores [10, 11], and divided by four components and by their respective dimensions (observed variables), are summarized in Table 1.

**Table 1:** Score of health-related quality of life variables of study population, divided by four components (life habits, social context, emotional status, and cognitive ability) and by their respective dimensions (observed variables)

		Well-being dimensions (Observed variables)	Sample	Median	IQR
Components	Lifestyle habits	Physical well-being	48.04 (10.01)	47.08	42.53–52.43
		Autonomy	45.51 (8.81)	45.17	40.08–50.77
		Financial resources	48.25 (9.78)	49.27	41.92–56.35
		Diet	2.25 (0.08)	2.00	1.00–4.00
		Physical activity	2 (0.03)	2.00	2.00–2.00
	Social context	Parent relations	49.79 (9.04)	49.50	44.09–54.65
		Peers	50.03 (9.96)	50.24	43.60–54.93
		School environment	49.14 (8.40)	48.61	43.81–54.22
		Bullying	48.03 (10.25)	48.07	38.29–58.84
	Emotional status	Psychological well-being	46.53 (10.82)	47.12	39.91–54.49
		Mood	47.23 (8.84)	47.15	41.21–54.02
		Self-perception	52.07 (10.16)	49.76	43.17–60.11
		Emotion	31.76 (8.08)	31.00	26.00–37.00
	Cognitive ability	Executive function	3.73 (2.18)	4.00	2.00–6.00
		School performance-Language & Literature	20.63 (5.01)	21.00	18.00–24.00
		School performance-Science	20.21 (5.24)	20.00	17.00–24.00
		School performance- Language acquisition	9.25 (3.15)	10.00	7.00–12.00

Data shown as mean (SD). IQR: interquartile range

In LH, physical well-being score was equal in comparison with the international T-value of reference group (mean 48.57-SD 9.64- median 47.08- IQR 42.53–55.60) [9, 17]. For autonomy, the group study mean was lower than a group of European boys aged between 12 and 18 years (reference group: mean 49.40- SD 10.6- Median 48.70- IQR 42.06–56.27). The international T-value for financial resources was similar in comparison to reference group values (mean 50.42- SD 9.80- median 49.28- IQR 44.18–62.86).

T-value of parent relations, peers, and school environment (SC component) was equal respect to the reference group. The dimension of bullying behaviours was lower than the threshold of the mean of the reference group (mean 50.94- SD 9.62- median 58.85- IQR 42.20–58.85). According to T-value in the psychological well-being and mood dimensions (ES component), our mean group was just below the threshold of the mean of the reference group. Self-perception score (body image, self-assurance, and self-esteem) was higher than the reference group (mean 48.28- SD 9.56).

In CA, the mean value of executive function dimension was 3.73 (SD 2.18). The descriptive value of school performance, language & literature and science, are reported in Table 1.

### Correlations among the well-being dimensions

Correlations among the variables were determined prior to verification of the hypothetical model (Table 2). The dimensions chosen for the composition of each component of the hypothesized health theory constructs were correlated with each other. A variable was considered redundant if the shared variance with another variable exceeded 50% (Pearson r > 0.71) [16]. All correlation coefficients among variables were less than 0.71; therefore, these results did not demonstrate multicollinearity [18].

**Table 2:**
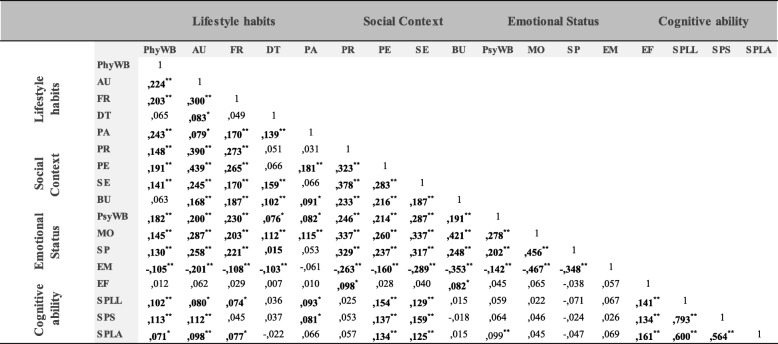
Correlations (Pearson’s correlation coefficient) between the score of well-being dimensions (observed variables).

*PhyWB* Physical Well-being, *AU* Autonomy, *FR* Financial resources, *DT* Diet, *PA* Physical Activity, *PR* Parent Relations, *PE* Peers, *SE* School Environment, *BU* Bullysm, *PsyWB* Psychological Well-being, *MO* Mood, *SP* Self-Perception, *EM* Emotion, *EF* Executive Functions, *SPLL* School performance-Language & Literature, *SPS* School performance-Science, *SPLA* School performance-Language Acquisition.

* *p* < 0.05; ** *p* < 0.01

### The measurement model

As described in Table 3, the four well-being components provided an acceptable explanation for their corresponding well-being dimensions, since all the coefficients were above 0.350 [19], with the exception of diet and physical activity in LH component, and executive functions in the CA component, with a coefficient < 0.350. Standardized regression coefficients, reported in Table 3, explain the contribution of each observed variables considered as predictor, to define the components. Thus, for LH component, the most important predictor was autonomy (β = .642, SE = .035, *p* < .0001), intended as the opportunity given to a child or adolescent to create his/her social and leisure time. The most important aspects in SC component were the relationship with the parents and the atmosphere in the child’s/adolescent’s home (β = .587, SE = .032, p < .0001), and the adolescent’s relationships with other adolescents (β = .544, SE = .034, p < .0001). For ES component, the mood observed variable was that with the greatest contribution (β = .776, SE = .026, *p* < .0001). Finally, school performance related to language & literature (β = .914, SE = .015, *p* < .0001) was the most important predictor in CA component.

**Table 3:** Standardized regression coefficient for the Structural Model

		Well-being dimensions (Observed Variables)	Standardized regression coefficient	Standard Error	P > |z|	Standardized regression coefficient [95% Conf. Interval]
Components	Lifestyle habits	Physical well-being	.366	.042	0.000	.282–.449
		Autonomy	.642	.035	0.000	.572–.711
		Financial resources	.489	.038	0.000	.414–.565
		Diet	.178	.044	0.000	.090–.266
		Physical activity	.242	.076	0.000	.227–.257
	Social context	Parent relations	.587	.032	0.000	.523–.651
		Peers	.544	.034	0.000	.477–.611
		School environment	.529	.034	0.000	.462–.595
		Bullying	.442	.036	0.000	.370–.513
	Emotional status	Psychological wellbeing	.406	.037	0.000	.331–.480
		Mood	.776	.026	0.000	.725–.828
		Self-perception	.594	.031	0.000	.532–.656
		Emotion	−.593	.031	0.000	−.654 - -.531
	Cognitive ability	Executive function	.169	.039	0.000	.086–.241
		School performance-Language & Literature	.914	.015	0.000	.882–.944
		School performance-Science	.867	.016	0.000	.834–.899
		School performance- Language acquisition	.659	.024	0.000	.610–.706

**Fig. 2:**
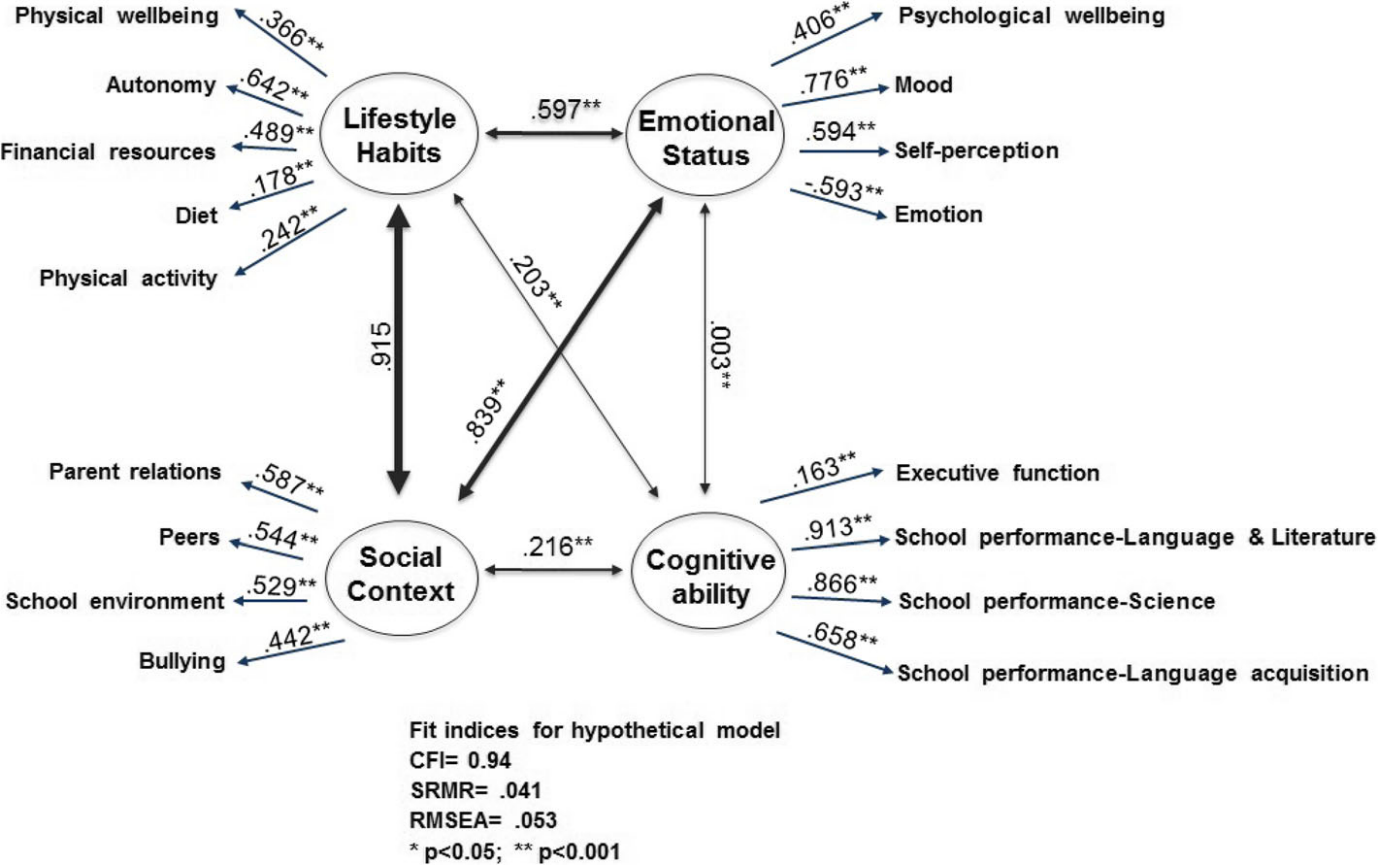
The Structural Model. CFI: Comparative Fit Index; SRMR: Standardized Root Mean Square Residual; RMSEA: Root Mean Square Error of Approximation

The standardized paths of the hypothesized health theory constructs from all the four components to their respective variables were specified in Fig. 2. The Structural Model Fit indices indicated that the proposed hypothetical model fits the data (χ2 = 2897.399 [df = 1119, *p* < .01], RMSEA = .053, SRMR was .041, and CFI = .94). The indices for hypothetical model showed that the measurement model fits adequately [20, 21].

As presented in Fig. 2, all standardized paths were significant except for the path between ES and CA (β = .0032, SE = .0476, *p* = 0.945). LH was positively associated with SC (β = .915, SE = .0463, *p* < .0001), ES (β = .597, SE = .050, p < .0001), and CA (β = .203, SE = .053, p < .0001). SC was positively associated with ES (β = .839, SE = .039, p < .0001) and CA (β = .216, SE = .050, p < .0001).

## Discussion

To the best of our knowledge, this is the first study that explores, through an integrated approach, the best indicators of health and well-being in adolescents, considering both the involvement of each variable of the different components affecting adolescents’ health (i.e. lifestyle habits, social context, emotional status, cognitive abilities) and the relationship between the four components.

Our results are in line with the International T-value of reference group for KIDSCREEN-52, obtained in a multicentre European study comprising a sample of 22,827 children recruited in 13 countries, except for variables belonging to emotional status and autonomy, in which our subjects exhibited lower score than the reference groups.

This could be partly attributable to some factors not evaluated in this study, including sleep quality, family socioeconomic status, and previous psychophysical condition.

The rationale to adopt an integrated approach for adolescence health and well-being is based also on the evaluation of successful individual functioning and positive social relationships.

Even though the variables considered in the present study have been previously described as influencing health status and well-being in adolescents [5, 6, 8], the integrated approach that allows a global vision of health (lifestyle, social context, emotional status, cognitive ability) is to be considered a new approach.

The main results can be summarized as follows: 1) the evaluation of the single variables within each component, autonomy, parental relation, mood and scholastic achievement, were the most determinant factors in the component definition; 2) the analysis of the relationship among the 4 components revealed the strongest relationship between LH and SC and between SC and ES.

### The four components in the assessment of adolescents’ health and well-being

Adolescence is considered the healthiest time of life, characterized by many of the requisite components of ideal health [4]. According to this perspective, a good health status is predominantly linked to four lifestyle habits (smoking status, body mass index, physical activity and diet), opening to different health programs in adolescents focused to maintain health behaviours [22].

However, epidemiological data showed that adolescence is a susceptible stage of life where other variables belonging to the psychosocial and mental skill components are matter of health status and well-being, potentially representing the early substrate of chronic degenerative diseases [4]. In line with this view, we have added psychosocial and mental skill components to the lifestyle determinants of health.

Therefore, we observed that autonomy, intended as opportunity in leisure time, and financial resources, had the highest weight among the LH variables. Leisure time has been shown to be associated with better life outcomes, scholastic achievement, and self-identity [23]. Also, it is thought to be important in order to provide the possibility for the promotion of new skills, the creation of social relationships, and new identities.

With regard to the role of financial resources, it has been well described a relationship with physical activity [24]. Data obtained from adolescents shows that the perception of living in low economical state will increase the general depressive symptoms. This relation can be due to same contextual risk factors as reduced social support, and risky health behaviour [25].

In the SC component, parents’, peers’, and schools’ support, exerted a positive effect on adolescents’ social environment [26]. Previous data showed that the quality of individual social relationships, such as between parents or peers, modifies the structure, function and development of social brain regions in adolescence [26]. However, among the different social settings, the family setting had the strongest involvement, providing the primary structure for transition to adult lives. This is in line with the fact that while social support from family is protective of adolescents’ healthy behaviours, friends’ support is usually associated with lower engagement in these behaviours [27].

For ES component, feelings such as loneliness, sadness, sufficiency/insufficiency, resignation, and self-perception are determinant. This suggests that positive or negative affective states and self-esteem are associated with emotional well-being and thus, with health status (i.e. life satisfaction) [28].

For CA definition, our results suggest that the most important variable is scholastic achievement, with particular focus on literature and scientific disciplines [17].

### Role of four components in the assessment of adolescents’ health and well-being

This study shows that LH and SC have the strongest bidirectional link on adolescents’ health and well-being, when compared to other components in our model. In general, large body of research supports the role of LH and SC factors in high healthy behaviours engagement [27], however far less is known about the potential interaction between these two components in influencing health status and well-being. Some studies have shown that healthy lifestyle behaviours are associated with better family functioning [27]. Vice versa, poor family condition is associated to an increased risk for unhealthy beahviors [29]. Also, adolescents with smoker parents have a greater chance of developing poor diet quality [30]. In our study, SC was also strongly connected with ES and this result can be explained by the fact that adolescence is a dynamic period characterized by rapid development in social-emotional behaviour [31]. Emotional responses and social settings interact and facilitate adaptive behaviour [8]. Although emotional development may find a psychological explanation in the social context, many studies focused mainly on biological factors that underlie adolescents’ emotional reactivity and regulation during social interactions, including hormonal changes and brain maturation [32, 33].

In particular, during adolescence there are significant changes in grey matter and white matter volumes in brain regions responsible for complex human behaviours, such as social relationships, mentalizing and self-related processing [34, 35]. The adolescent interprets social and emotional cues, modulating his own affective responses thus, the social brain develops in connection with emotions [36]. Our results support the principle that social relationships modulate neural networks involved in affective behaviour [37].

Resilience, defined as the ability to bounce back or recover after difficulty/hardship, can support the role of the social setting on the emotional component during adolescence [38]. In fact, high resilience in social support from family is associated with a lower level of depressive symptoms [39]. In addition, social reinforcement from friends is the strongest protective factors against anxiety behaviours and depression [40].

Meanwhile, ES component showed a statistically bidirectional link with lifestyle habits. In healthy conditions, this relation is poorly known, while great amount of research focused on the association between unhealthy lifestyle factors, mainly diet and physical activity, and symptoms of anxiety and depression [41]. Moreover, many adolescents may learn to deal with emotional problems by eating unhealthy food, using lifestyle behavioural strategies in order to manage affective reactivity [42].

Based on our results, LH and SC were equally linked with CA. In this regard, a body of literature showed that adolescents’ perceived social support, from family, peers, teachers, was associated with higher scholastic achievement [43]. Dubow and colleagues demonstrated that students’ report of aggregated and interactive social support from family, peers, and teachers, predicted their achievement two years later [44]. In spite of the studies linking social context to scholastic achievement, little is known about the mechanisms potentially explaining these relations. Probably, in our study, the strong association between SC and ES may contribute to achievement indirectly by means of motivational and affective outcomes.

### Strengths and limitations

This study included a large number of known variables in order to assess throughout an integrated approach, the best indicators of health status and well-being in adolescents. However, certain limitation of the study should be acknowledged. First, given the reliance on self-report data, it would have been beneficial to conduct focus groups with students, to explore concepts in more depth. Secondly, since the questionnaires were completed during a school class, it is possible that the school classroom environment may have biased the students’ responses. Moreover, given the results related to the perception of the parental relationship and its link with lifestyle habits, it might be particularly interesting to examine the socioeconomic family status. Also, we did not include parameters such as sleep quality questionnaire that is usually associated with parental quality of life and well-being. In addition, for cognitive assessment, as reported in literature, we have chosen only Stroop test for its application in attention, processing speed, cognitive flexibility, and working memory evaluation [45]. Furthermore, we did not assess the potential impact of gender difference.

### Implications and future research

Preventive interventions in healthy adolescent population is useful to improve resilience, happiness, social involvement, self-esteem and sociability, in order to reduce potential risk factors. In this perspective, the integrated approach proposed here is highly flexible and adaptable in its potential applications. This approach would help teachers to select more appropriately personalized educational programs aiming to monitor their compliance and effectiveness. Moreover, evaluation of the best indicators of health and well-being in adolescents represents an important goal in primordial prevention field in order to identify the needs and try to meet them in a more active way. Thus, the possibility of using this integrated approach might be of public health relevance in which a network of different stakeholders dedicated to adolescent education may cooperate together to increase awareness, change behaviour, and create environments that support good health practices.

There are two major issues for future research. First, more studies measuring health and well-being objectively or at least a wide range including other parameters linked to social environment are needed. Second, more longitudinal studies are necessary to clarify the direction of causality between single variables and components of adolescent’s health and well-being.

## Conclusion

Based on the aforementioned findings, the proposed approach suggests the importance of an integrated perspective to characterize adolescents’ health status and well-being. The results highlight the role of different components, life-style, social, emotional, and mental ones, according to a multifactorial and multisectorial approach. The integrated approach suggested here may highlight additive synergistic effects of the various components in health and well-being assessment that would be overlooked if a single factor approach was used.

## Data Availability

The datasets used and/or analysed during the current study are available from the corresponding author on reasonable request.

## Abbreviations

AVATAR: A new purpose for promotion and eVAluation of healTh and well-being Among healthy teenageRs
BMI: Body Mass Index
CA: Cognitive abilities
CFI: Comparative Fit Index
ES: Emotional status
LH: Lifestyle habits
RMSEA: Root Mean Square Error of Approximation
SC: Social context
SD: Standard deviation
SEM: Structural equation modelling
SRMR: Standardized Root Mean Square Residual
